# Supplementary material for: Exercise Normalized the Hippocampal Renin-Angiotensin System and Restored Spatial Memory Function, Neurogenesis, and Blood-Brain Barrier Permeability in the 2K1C-Hypertensive Mouse
Source: Int J Mol Sci. 2022 May 16;23(10):5531. doi: 10.3390/ijms23105531 (PMC9146761; doi:10.3390/ijms23105531)
Supplement: Supplementary file 1 [file ijms-23-05531-s001.zip › ijms-1701550-supplementary.pdf]

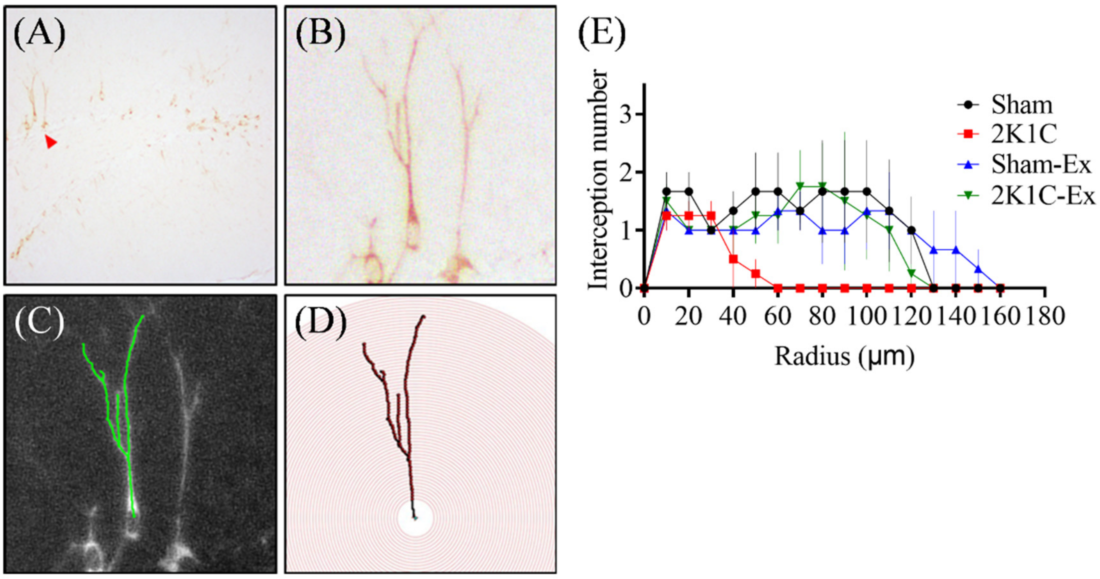

Suppl Figure S1. Exercise restores the dendritic complexity of dentate gyrus in 2K1C-hypertensive mouse model. (A-D) The schematics for Sholl analysis procedure. (**E**) Quantitative results of the number of dendritic intersections with concentric rings with increasing diameters of 10 µm (Sholl analysis). The sample sizes for sham, 2K1C, Sham-Ex, and 2K1C-EX are 3, 4, 3, and 4, respectively.

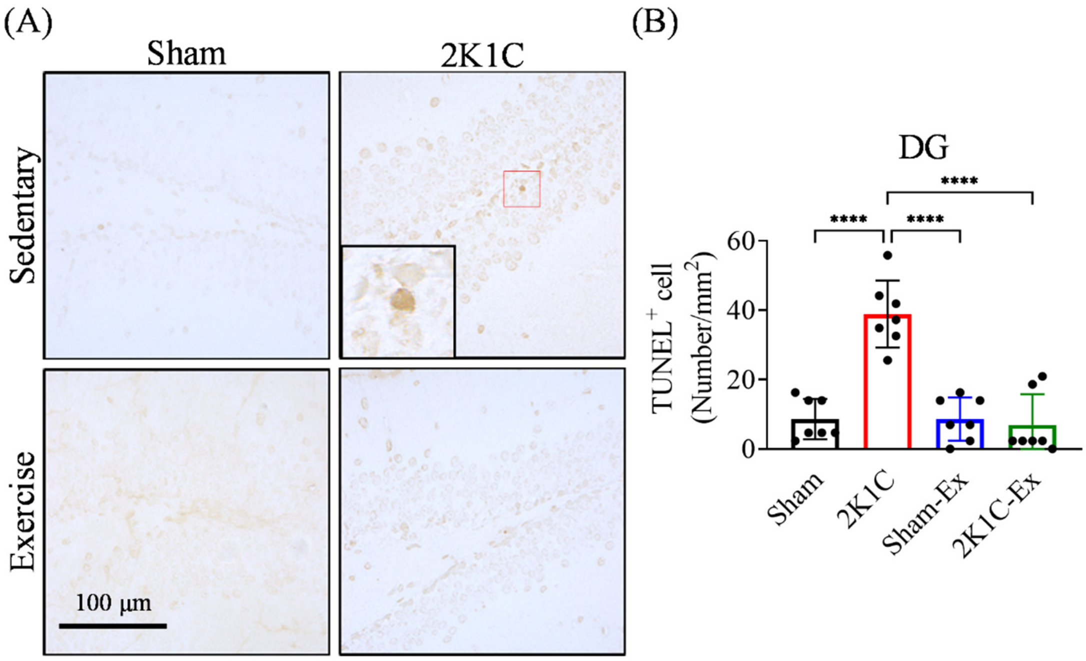

Suppl Figure S2. Exercise prevents the 2K1C-hypertension-induced neuronal cell apoptosis of the dentate gyrus. (**A**) The Tunel staining micrographs for dentate gyrus (DG) of hippocampal. (**B**) The quantitative result for apoptosis in the DG. The averaged data and s.d. are plotted. The statistical analysis was performed by one-way ANOVA and holm-sidak's multiple comparisons. The sample sizes are 7 for each group. ****: $p < 0.0001$. Scale bar, 100 μm. The Tunel positive neuron has been enlarged in (**A**).

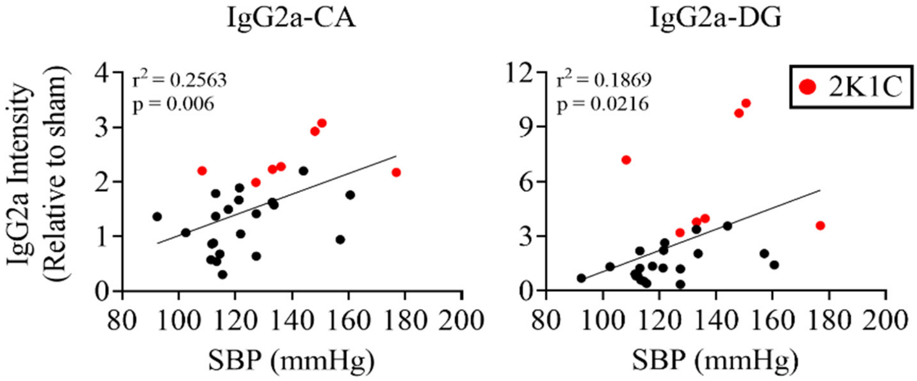

Suppl Figure S3. Systolic blood pressure is correlated with IgG leakage levels in the CA and DG. The scatter plots of the correlation between the IgG2a leakage and systolic blood pressure. The 2K1C group individual points have been labeled in red color.

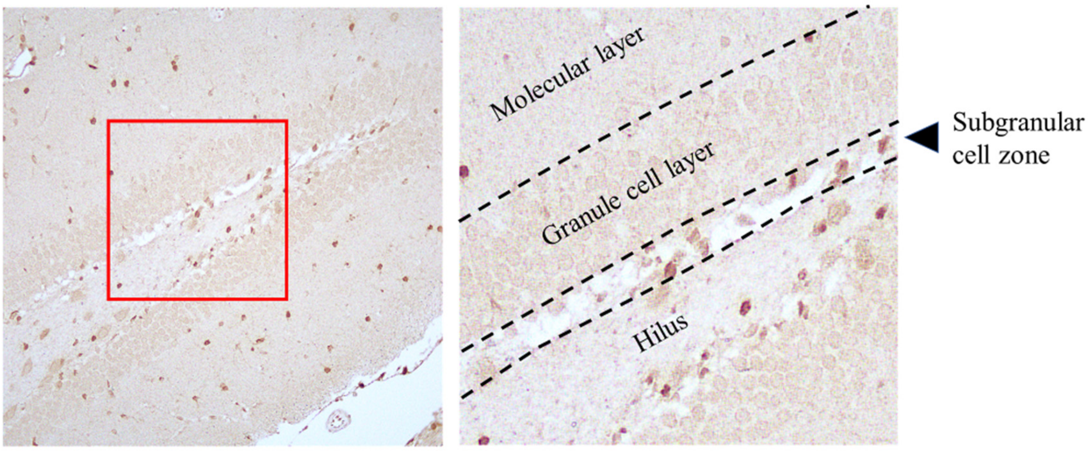

Suppl Figure S4. The Ki67$^+$ cells in the subgranular cell zone. Left panel, a 20x objective lens acquired image for Ki67 IHC staining. Right panel, the different layer of dentate gyrus.

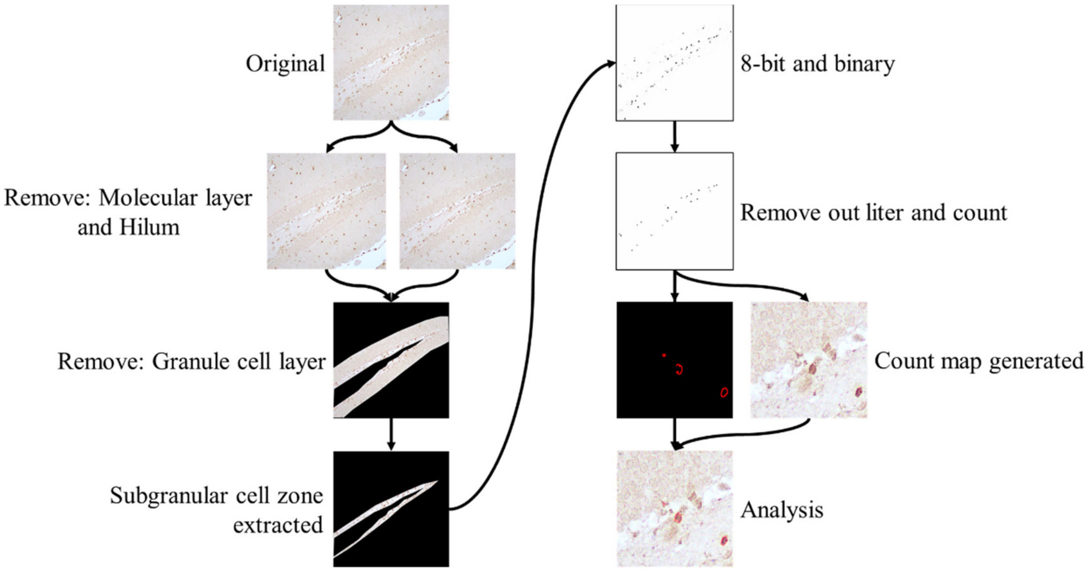

Suppl Figure S5. The Ki67$^+$ cells quantification flowchart. The molecular layer, hilus, and granule cell signal would be removed from origin image in the first step. The removed part would fill up with background color. Next, we generated the 8-bit picture and binary map by signal threshold. Before the particle analysis, we removed the outlier signal according to size and shape discerption. After the count map was generated by particle analysis, we merged the count map with the original picture and counted the actual neural stem cell numbers. Only the cell with an intact soma shape would be counted.
